# Sunlight-driven eco-friendly smart curtain based on infrared responsive graphene oxide-polymer photoactuators

**DOI:** 10.1038/s41598-018-21871-3

**Published:** 2018-02-27

**Authors:** Leeladhar, Parul Raturi, J. P. Singh

**Affiliations:** 1 Department of Physics, Indian Institute of Technology Delhi, Hauz Khas, New Delhi, 110016 India; 2 Solid State Physics Laboratory, Lucknow Road, Timarpur, Delhi, 110054 India

## Abstract

Photomechanical actuation is the conversion of light energy into mechanical energy through some smart materials. Infrared-responsive smart materials have become an emerging field of research due to easy availability and eco-friendly nature of their stimulus in the form of sunlight, which contains about 50% of near-infrared(nIR) making these materials useful at macro-scale photoactuator applications. Here, we demonstrate fabrication of highly versatile nIR triggered photoactuators based on graphene oxide/polycarbonate bilayers that offers fast, low-cost fabrication, large deflection, reversible actuation and wavelength-selective response. The photoactuators are realized by vacuum filtration of graphene oxide/water dispersion through polycarbonate membrane resulting graphene oxide/polymer bilayer structure. The photoactuation response was measured in the form of deflection from equilibrium position as a result of infrared-irradiation. The deflection is caused by the generated thermal stress at the interface of bilayers due to mismatch of thermal expansion coefficient as a results of nIR absorption by graphene oxide and subsequent temperature rise. A maximum deflection of 12 mm (circular-shaped structure with diameter 28 mm) with corresponding bending curvature of 0.33 cm^−1^ was shown by this photoactuator for illumination intensity of 106 mW/cm^2^. Few applications of these photoactuators such as sunlight-driven smart curtain, infrared actuated curtain and self-folding box are also demonstrated

## Introduction

Sunlight is a renewable and eco-friendly source of energy, so it is always desirable to utilize this energy for the use of mankind. There are various mechanisms to convert this energy into useful form and photoelectric effect is one of these. However, in recent times, photomechanical effect, which is a process of conversion of light energy into mechanical energy by using some smart materials has received much attention. Smart materials are capable of changing their shape or dimensions under external stimuli and these are widely used in actuation devices. There are plenty of smart materials which respond to heat^1^, light^2,3^, electricity^4,5^, and magnetism^6^ but recently the thrust area of research on actuators is in the fabrication of photomechanical actuators based on carbon nanostructures/polymer hybrid materials^7–34^ because of their potential applications in soft robotics^35^, drug delivery^36^, photo actuated motor^37,38^, adaptive micro-mirror^39^ and artificial muscles^40^. Photomechanical actuation offers a lot of advantages over traditional devices, including wireless actuation, electromechanical decoupling (and therefore low noise), electrical circuit elimination at point of use, and massive parallel actuation of device arrays from single light source^41^. There are a number of photo-responsive polymer materials such as azobenzene^42^, spiropyran^43^, diarylethene^44^ in which ultra-violet (UV) irradiation induced isomerization and reaction causes the actuation. However, the use of UV as a stimulus source for such materials restricts their use in biological applications because of the inevitable damage to the biological tissues. Alternatively, polymeric hybrid structures in which infrared light absorbing carbon nano-structured materials i.e. graphene/graphene oxide are integrated with polymer materials can show promising application as photomechanical actuators. Such materials can also be used in biological system due to the good penetration and biocompatibility of near–infrared (nIR) light as a triggering source. Among the various type of nIR responsive nanostructures/polymeric hybrid materials for photoactuation application, graphene and graphene oxide (GO) based materials have become more prominent in modern times, due to their cost-effectiveness and ease of fabrication along with extraordinary mechanical, thermal and optical properties^45,46^. However, film formation by vacuum filtration of graphene oxide is much easier than graphene due to their hydrophilic and hydrophobic nature, respectively. In addition, thermo-mechanical property i.e. coefficient of thermal expansion (CTE) value of graphene oxide in ambient conditions is more negative than graphene which is desirable to show large photoactuation. While graphene oxide shows decreasing absorption from visible to nIR^47^, it shows an excellent photothermal conversion efficiency in the band of nIR^48^. When it is integrated with some polymer material to form a hybrid structure, it will absorb nIR light and convert into thermal energy like a nanoscale heater, which can cause temperature rise of the hybrid structure resulting in the functionalities like photoactuation^49^.

Recently, some work has been done on GO based photoactuators in which GO/poly(vinylidenefluoride-hexafluoro propylene)(PVDF-HFP) nanocomposite films were fabricated by directly casting a mixed solution of PVDF-HFP containing homogeneously dispersed GO. It has shown visible light powered tumbler motion^19^. In another report, researchers have shown fabrication of GO-poly (*N*-isopropyl acrylamide) nanocomposite hydrogels and their bending/unbending behavior upon IR irradiation^49^. However, time taken to switch from normal state to the bending state is comparatively large of the order of few seconds. Moreover, the fabrication process of such photoactuators require complex and time consuming method of mixing multiple component materials to form nanocomposites. In a recent research, Cheng *et al*. have fabricated single layer GO films with asymmetric surfaces on both the sides^25^. This film was then used for actuator application by multiple stimuli like humidity and light. A similar study was also done by Chen *et al*. in which they have fabricated multi-responsive GO/BOPP bilayer actuators triggered by humidity and IR light^50^. In another interesting study, Hu *et al*. have developed RGO-CNT/PDMS bimorph based tubular shaped photoactuator^23^. However, it is important to note that the photoresponse of all such actuators was shown only under artificial source of infrared light i.e. IR lamp/solar simulator rather than natural and eco-friendly energy source like sunlight which may limit their applications at macro scale.

The present research work demonstrates a novel approach of integrating graphene oxide with polycarbonate membrane to form a bilayer structure through vacuum filtration technique. The polycarbonate membrane was chosen due to its large positive value of thermal expansion coefficient of 65 ppm/K^37^. The process is very simple, cost effective, scalable and fast. It also does not require the route of nanocomposites fabrication through mixing of multiple components, which has been used in most of the earlier research work done on GO based photoactuators. The photoactuator comprised of GO/polycarbonate bilayer structure has been realized and its actuation response was measured in the form of deflection from equilibrium state upon IR illumination. The maximum deflection was found to be 12 mm within 3 s with corresponding bending curvature value of 0.33 cm^−1^ (Note S1) for 106 mW/cm^2^ as intensity of illumination. The effect of light illumination intensity on photoactuation response was also studied. Finally, these photoactuator structures were used under real sunlight to demonstrate applications such as sunlight-driven smart curtain and IR actuated self-folding box. These structures may also find potential applications in micro soft robotics and controlled drug delivery systems.

## Results and Discussion

**Figure 1:**
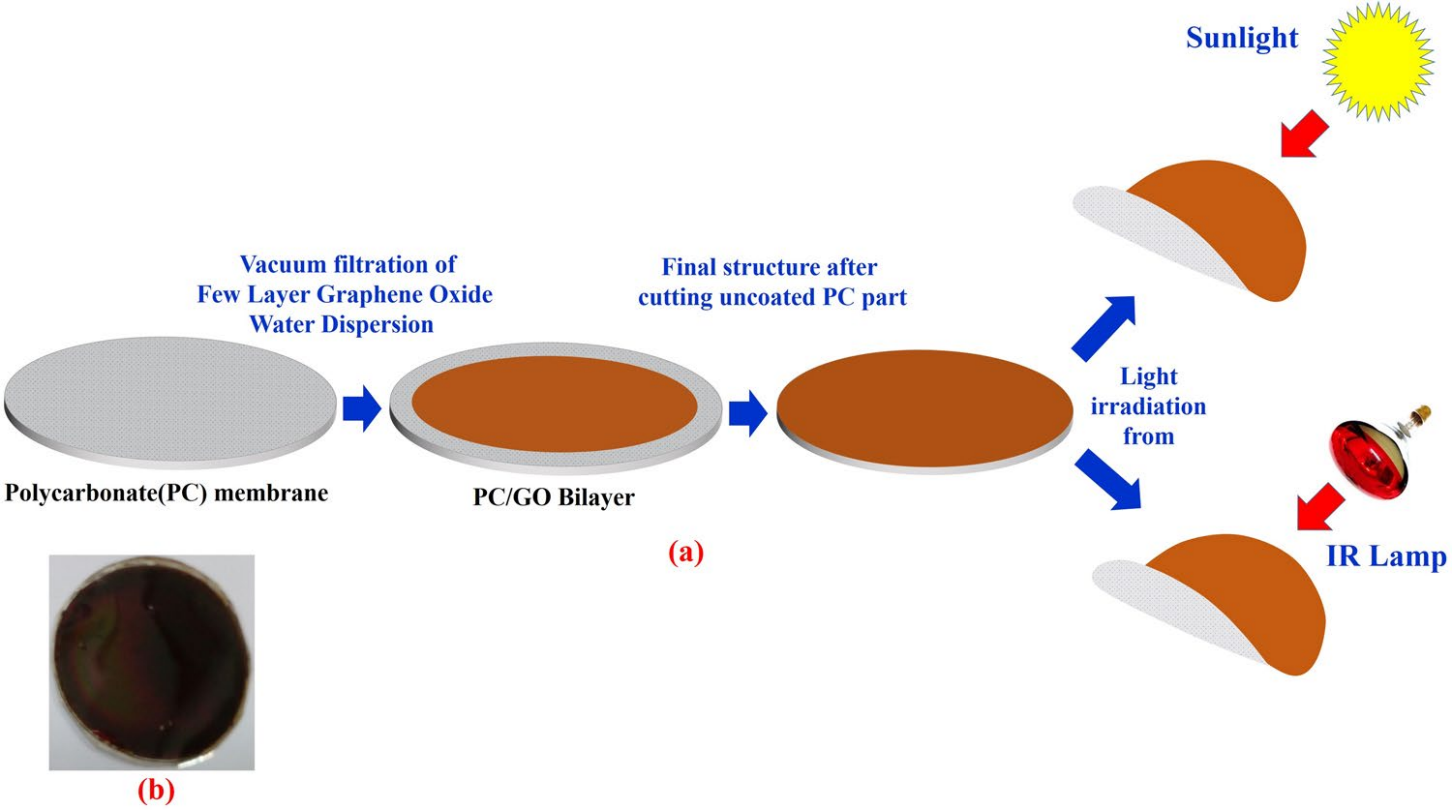
(**a**) Schematic illustrating the fabrication of Graphene Oxide/Polycarbonate bilayer photoactuator and its actuation under infrared and sunlight illuminations. (**b**) The optical image of the fabricated GO/PC bilayer structure. *Comment: The first author has taken the image of IR lamp.

The schematic of fabrication process for GO/PC bilayer photoactuator is shown in Fig. 1. The vacuum filtration was used to filter 1 ml of GO water dispersion through polycarbonate membrane followed by drying in ambient to form GO/PC bilayer structure. The average thickness values of PC and GO film were found to be 22 µm and 8 µm respectively as shown by cross-sectional SEM image (Fig. S1). The extra part of PC membrane without GO coating was cut away to make curtain structure with diameter of about 28 mm.

**Figure 2:**
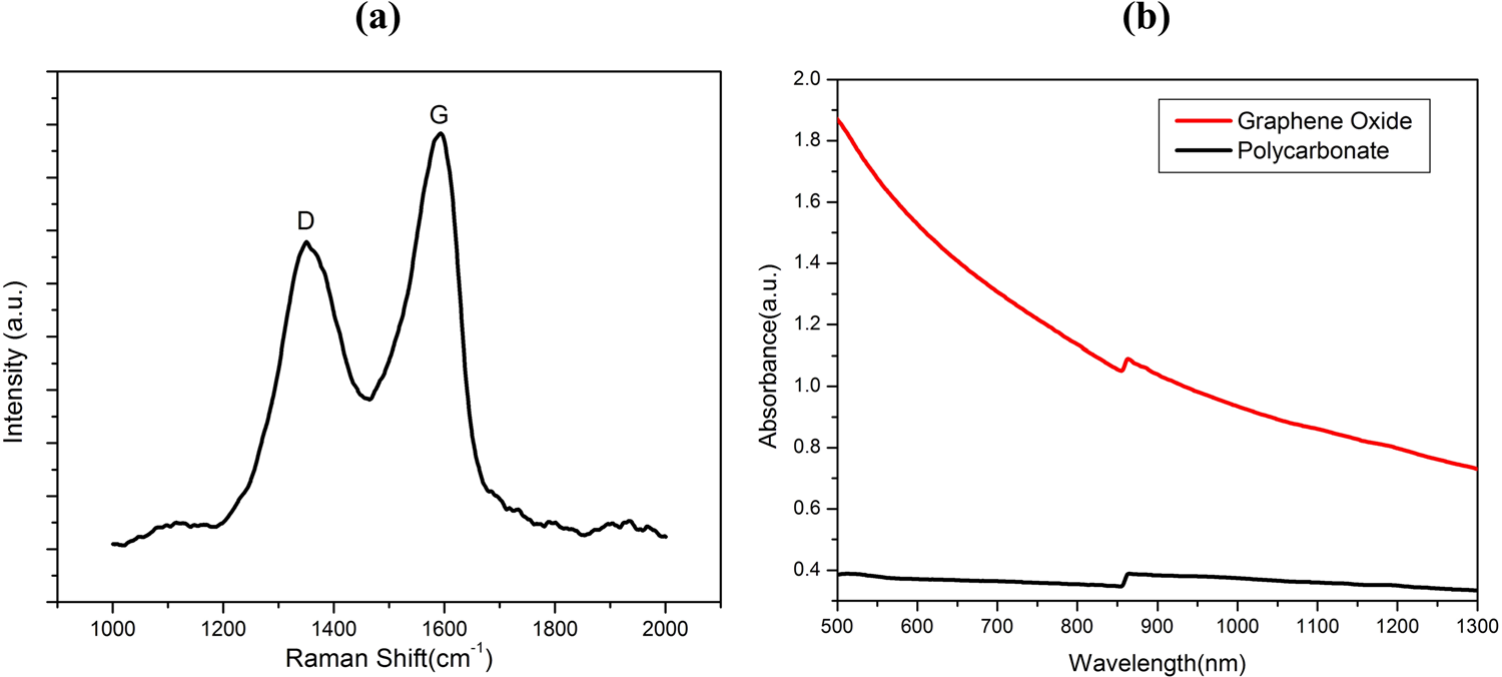
(**a**) Raman spectrum of graphene oxide layer on PC membrane. (**b**) Vis-NIR spectrum of PC membrane and graphene oxide.

The Raman spectrum of GO film is shown in Fig. 2(a). Two prominent characteristic peaks were obtained at 1360 cm^−1^ and 1592 cm^−1^ values of Raman shift. These peaks correspond to D and G bands of GO, respectively. The D band is associated with the disordered graphene edges and G band is the result of the first order scattering of E_2g_ mode of sp^2^ carbon domains. The shape and intensity of D and G peaks show that GO used in the fabrication of this actuator is few layer graphene oxide. As graphene oxide absorbs IR light^49^, so IR absorption study was conducted for GO film and PC membrane as shown by Vis-NIR spectrum in Fig. 2(b) which shows a plot of absorbance versus wavelength. It is clearly visible from this plot that GO film exhibits much larger absorbance than PC membrane which may be helpful for temperature rise required in photoactuation.

**Figure 3:**
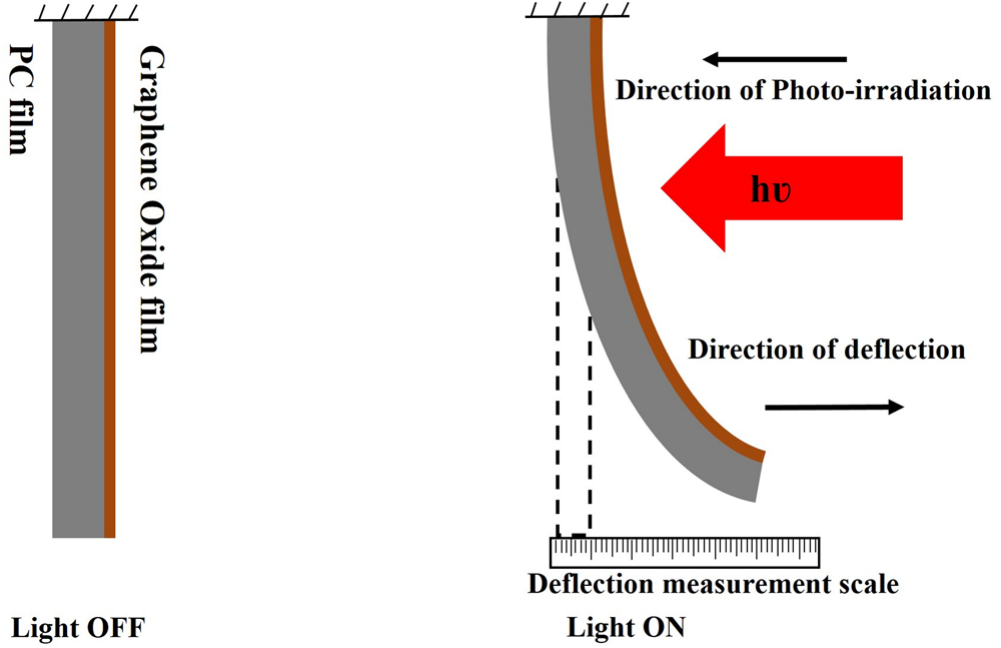
Schematic of actuation measurements of GO/PC bilayer photomechanical actuator under infrared light illumination.

**Figure 4:**
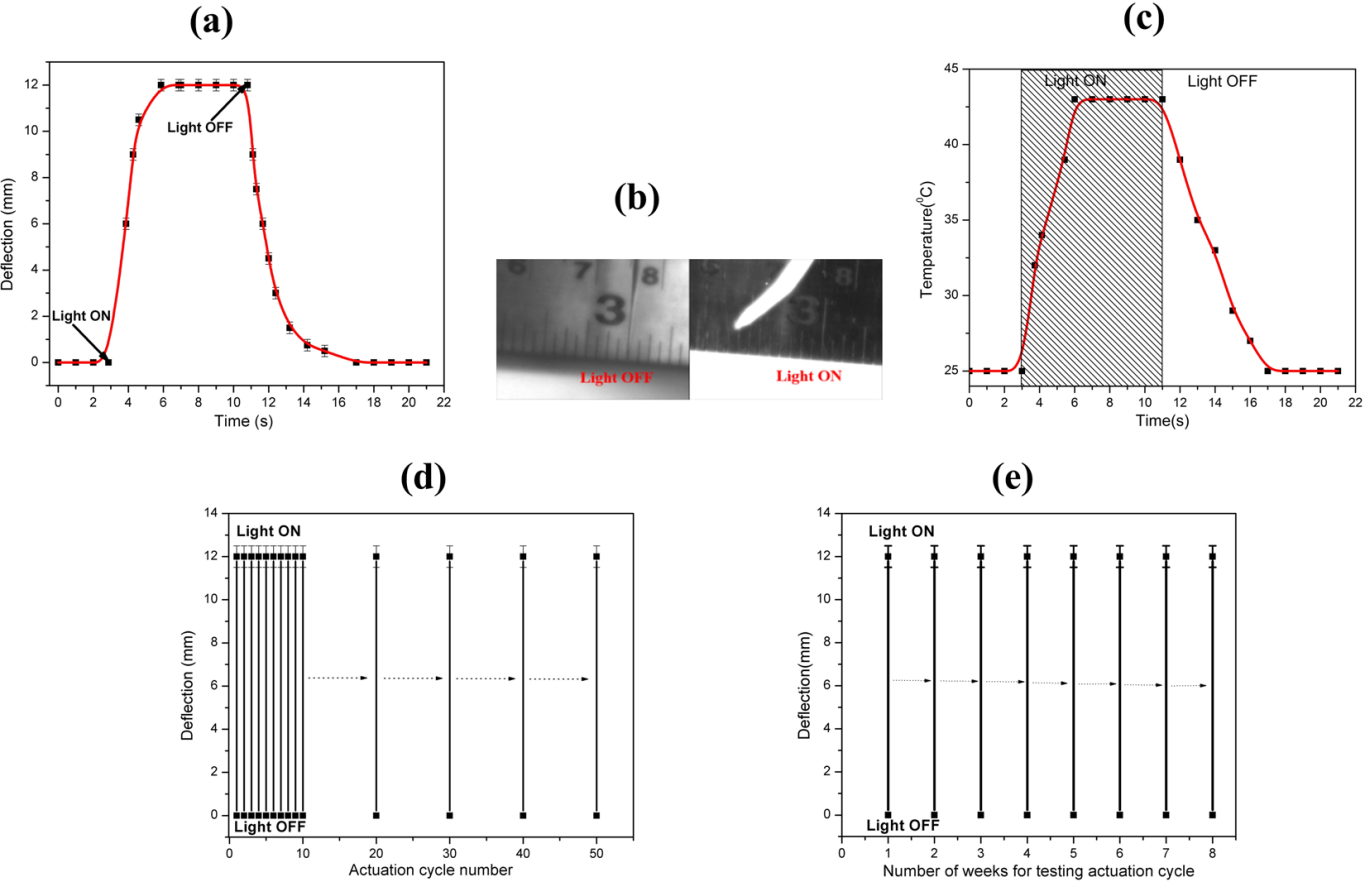
(**a**) Variation of deflection of GO/PC bilayer photoactuator from its equilibrium position with time after IR light of intensity 106 mW/cm^2^ was turned ‘ON’ and ‘OFF’. (**b**) Photos of GO/PC bilayer photoactuator, equilibrium state when IR light was ‘OFF’ and the maximum deflected state when IR light was ‘ON’ at 106 mW/cm^2^ as illumination intensity. (**c**) Temperature variation of GO face of GO/PC bilayer photoactuator during actuation cycle illuminated with IR light having intensity value of 106 mW/cm^2^. (**d**) Reversibility of actuation of GO/PC bilayer photoactuator. (**e**) Long-term actuation reliability studies of GO/PC bilayer photoactuator.

Schematic diagram for the measurement of photomechanical actuation of GO/PC bilayer is shown in Fig. 3 wherein, the circular actuator structure was fixed along one of its diameter end. IR light was illuminated on GO face resulting its deflection from equilibrium position towards the light source, which was then measured using a fixed deflection measurement scale. Figure 4(a) exhibits the photomechanical actuation response of GO/PC bilayer structure (Movie S1). It illustrates the variation of deflection from its equilibrium position as shown in Fig. 4(b) with IR light illumination time for a fixed light intensity value of 106 mW/cm^2^. As IR lamp was switched ‘ON’ and light was allowed to fall on the GO face of GO/PC bilayer circular photoactuator fixed at one of its diameter end, then the bilayer structure starts deflecting from its equilibrium position very quickly i.e. with a response time of less than 1 s. The deflection then increases with time and attains its maximum value i.e. 12 mm within 3 s as shown in Fig. 4(b). This shows a much faster response as compared to the earlier reported GO nanocomposites based photoactuators which is of the order of few seconds^49^.

The GO/PC bilayer photoactuator is comprised of two layers of different materials with difference in their values of Young’s modulus and coefficient of thermal expansion/contraction. When such layers are not bonded and undergo a temperature change, this results in the different values of strain in these individual layers; but in bonded condition any temperature change brings misfit strain and hence, generate internal thermal stress. So, deflection of this photoactuator is caused by the generation of such thermal stress at the interface of bilayers as a result of IR absorption and excellent photothermal effect of GO. The GO layer acts like nanoscale heater such that absorbed infrared light energy is converted into thermal energy which results in the temperature rise in GO layer of 18 °C for light intensity value of 106 mW/cm^2^ as shown in Fig. 4(c)^49^. However, Kotov *et al*. reveled that negative CTE of GO is in fact pseudonegative thermal expansion which is due to thermohydration effect and intrinsic CTE was found to be close to zero at very low humidity of about 1%^51^. But in ambient conditions GO exhibited negative CTE value of −50 ppm/K^52^. So the temperature rise in GO layer causes its contraction. The thermal energy is also transferred to the PC layer of this photoactuator causing its expansion because of its positive CTE value of 65 ppm/K^37^. Therefore, the opposite trend in terms of thermo-mechanical properties i.e. CTE values of the individual layers bring a large mismatch of thermal expansion coefficients(CTE) in this GO/PC bilayer structure along with IR absorption property of GO layer results in the fast and large photomechanical response of this photoactuator. The moment when IR lamp was put ‘OFF’, the deflection starts decreasing and becomes almost zero within 5 s. This process was tested to be reversible for more than fifty consecutive cycles as shown in Fig. 4(d). The reversibility in actuation of this bilayer actuator is imparted by the excellent mechanical property of GO layer. As we have proposed one of the application of this photoactuator as sunlight-driven smart curtain so its long term actuation reliability is highly essential. This study has been done as shown in Fig. 4(e), wherein the GO/PC bilayer photoactuator was tested to be stable in its actuation performance for consecutive eight weeks which shows its long term reliability. This structure was also found to be rugged in terms of its physical conditions as there was no observation of problem like delamination of bilayers after long term use which is desirable for the applications like smart curtain.

**Figure 5:**
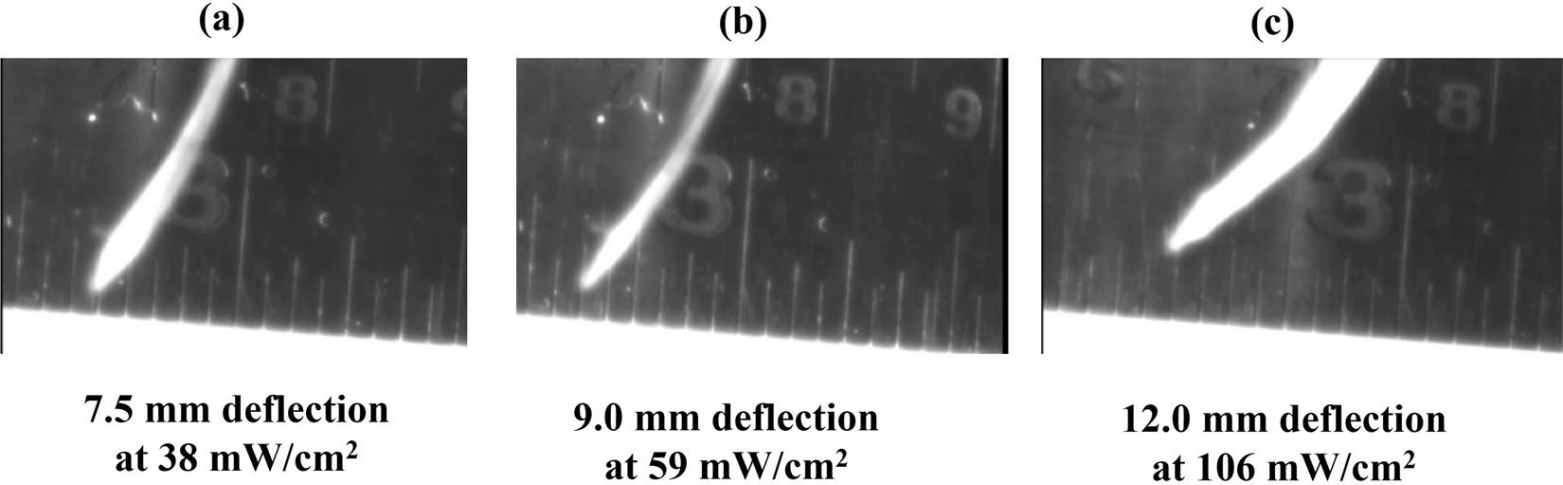
Photoactuation response of GO/PC bilayer photoactuator showing the maximum defection at IR light illumination intensity values of (**a**) 38 mW/cm^2^, (**b**) 59 mW/cm^2^, and 106 mW/cm^2^.

The photomechanical actuation response and hence, the value of maximum deflection were also determined for different values of the IR light intensity. The higher illumination intensity brings higher temperature rise due to the more IR absorption by the actuator surface and which was found to be 10.4 °C, 12.8 °C and 18 °C corresponding to the IR light intensity values of 38, 59 and 106 mW/cm^2^, respectively. The maximum deflection were found to be 7.5, 9.0 and 12 mm corresponding to the IR light intensity values of 38, 59 and 106 mW/cm^2^, respectively as shown in Fig. 5.

### Infrared and sunlight-driven smart curtain

**Figure 6:**
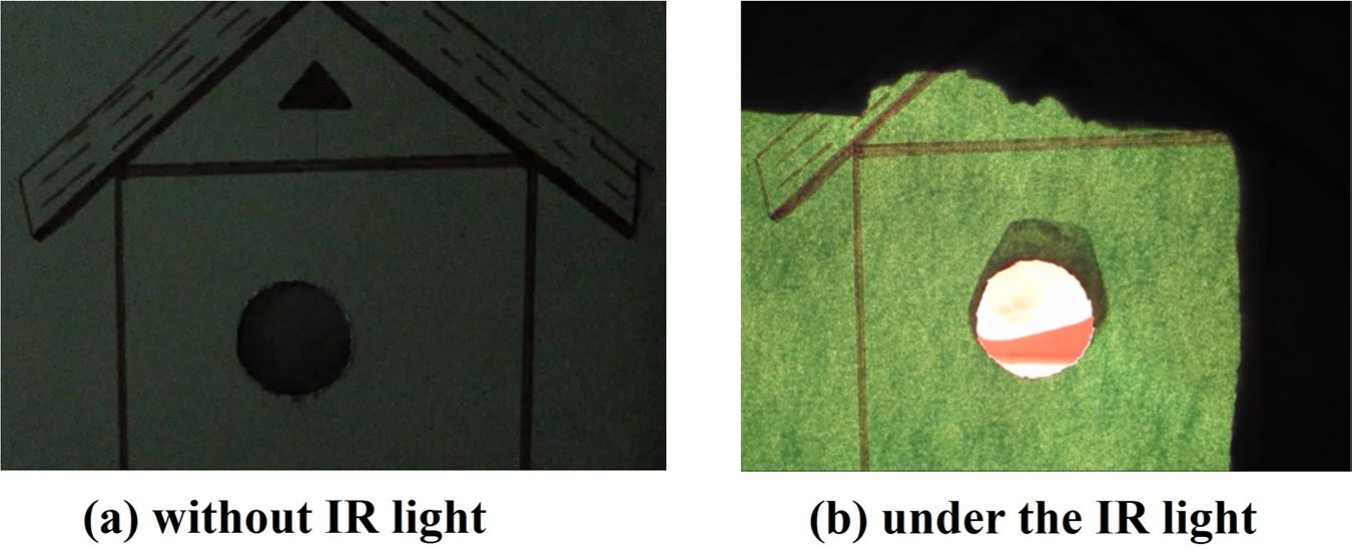
GO/PC based curtain actuated by infrared (IR) light. (**a**) Closed state without exposure of IR light. (**b**) Open state with exposure of IR light.

A prototype experiment was performed using this fabricated GO/PC bilayer photoactuator to demonstrate its practical applications as IR actuated as well as sunlight-driven smart curtain for eco-friendly and energy efficient windows. GO can absorbs IR light and is high-efficient photothermal conversion material which can act like heating layer in GO/PC bilayer actuator^49^. As the sunlight contains about 50% of nIR^53^ and hence, can act as natural and eco-friendly source of infrared energy which can be used as a stimulus for GO/PC bilayer photoactuator. So the use of real sunlight absorption by GO to show photothermal actuation can open new ways for sunlight energy harvesting in the applications such as smart curtain. A house model was made using a hard paper having circular window of diameter 26 mm. The actuator structure was fixed with one of its diameter end on circular window of house model such that it covers the window like a curtain as shown in Fig. 6. The curtain was then illuminated first with IR light from an IR lamp at intensity of 106 mW/cm^2^. Interestingly, curtain gets open under IR light ‘ON’ condition and closes when IR light was put ‘OFF’ (Movie S2). The close/open states of the curtain without IR and under IR light are shown in Fig. 6(a,b), respectively.

**Figure 7:**
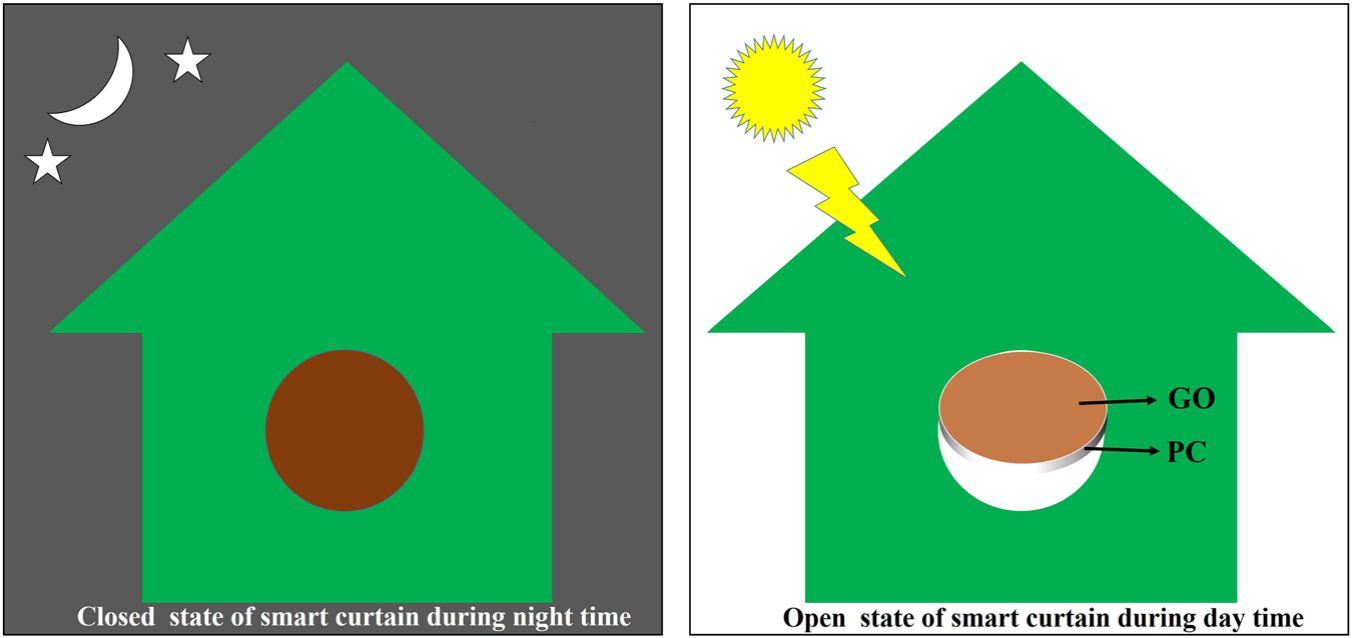
Schematic illustrating the operation of GO/PC bilayer photoactuator based smart curtain (view captured from outer side of the house).

**Figure 8:**
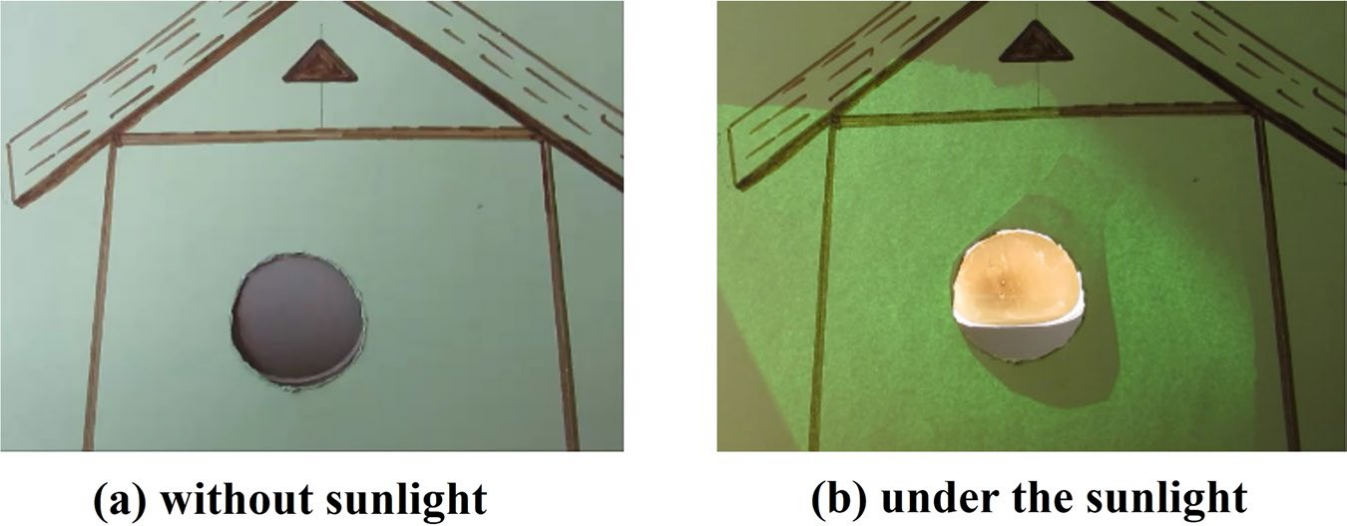
A prototype GO/PC bilayer smart curtain (view captured from inner side of the house). (**a**) Closed state without sunlight. (**b**) Open state under the sunlight with intensity of 100 mW/cm^2^.

This structure was also characterized under sunlight for the various parameters like deflection, reversibility and long term reliability. It was found that the actuator shows almost similar performance as it shows under the IR lamp with same light intensity. The same structure was then used to demonstrate sunlight-driven smart curtain (Movie S3). A curtain can be considered as the smart curtain if it gets open during the day time under sunlight automatically without using any external power source and the sunlight enters inside the house and closes by itself during night time as shown by the schematic in Fig. 7. When this GO/PC bilayer curtain structure was exposed to sunlight then it gets open automatically and closes when sunlight was blocked from falling over it. The curtain’s actuation performance under sunlight was tested to be reversible for more than fifty cycles to ensure its reliability. The Fig. 8(a,b) shows the close/open state of sunlight-driven smart curtain without sunlight and under sunlight conditions, respectively. In order to realize eco-friendly and energy efficient windows such sunlight-driven smart curtain may be used in place of existing technology, which needs power for their operation.

### Infrared actuated self-folding box

**Figure 9:**
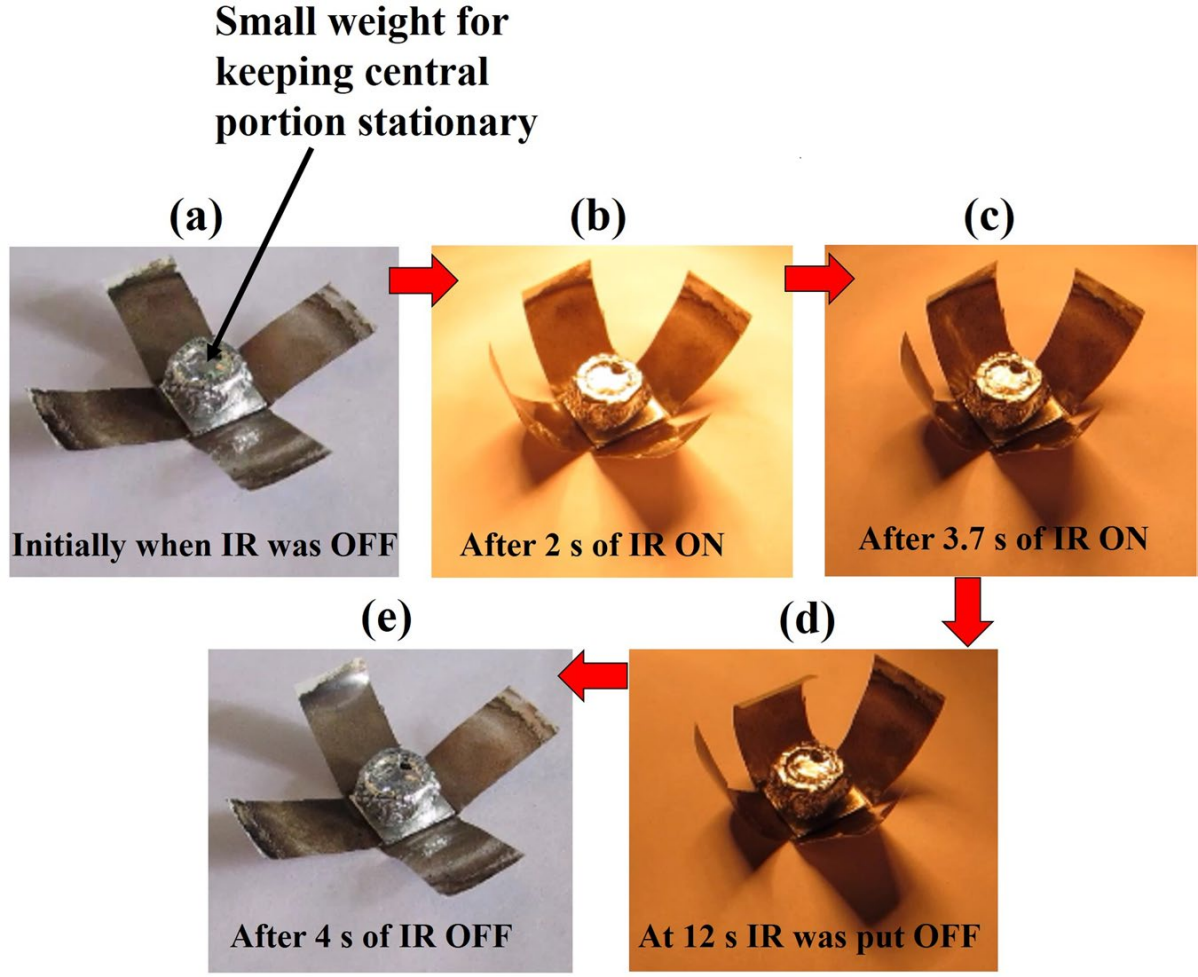
Actuation cycle of infrared triggered self-folding box based on graphene oxide/polycarbonate bilayer structure.

Another application of the fabricated GO/PC bilayer structure was demonstrated as an infrared actuated self-folding box, which may find potential use in soft-robotics. The self-folding box consists of plus sign shaped structure made up of GO/PC bilayers with central portion having dimensions as 1.0 cm × 1.0 cm and four wings having dimension of 1.0 cm × 1.5 cm. The complete actuation cycle of self-folding box is shown in Fig. 9 (Movie S4). Initially at *t* = 0 s, IR light was turned ‘OFF’ and the box has a structure as shown in Fig. 9(a). When IR light was put ‘ON’ with an intensity value of 106 mW/cm^2^, the wings of the structure started moving upwards and just in 2 s, it acquired the box like shape which then transformed into its final box like shape in 3.7 s as shown in Fig. 9(b,c). Finally, when IR light was put ‘OFF’, the box again regained its initial shape within just 4 s as shown in Fig. 9(d,e), which shows a very quick transformation from final state to initial state as compared to the earlier reported work of similar kind^27^. This fast recovery response may be imparted due to the opposite trends in thermo mechanical properties and hence large CTE mismatch of individual layers of GO/PC bilayer photoactuator structure.

In conclusion, we have investigated the photomechanical actuation behavior of graphene oxide/polycarbonate bilayer structure. The actuation was measured in the form of the deflection from its equilibrium position upon IR irradiation, which increases with time and gets saturated to a maximum deflection value. The actuation shown by this bilayer actuator is found to be fast and reversible in nature. The response time is less than 1 s and it takes only 3 s to attain maximum deflection state from its equilibrium state. The maximum deflection increases with increase in IR light illumination intensity. Few prototype applications such as sunlight-driven smart curtain and IR actuated self-folding box using GO/PC bilayer photoactuator have been demonstrated. The materials used in the fabrication of this bilayer actuator can also be used to realize other potential applications such as in soft robotics, controlled drug delivery, opto-mechanical bolometer for imaging and radiation measurement devices for nuclear reactors.

## Methods

### Materials

GO water dispersion was purchased from Log 9 Materials with concentration of 2 mg/ml. The polycarbonate membrane (Millipore, 0.2 µm pore size, diameter 47 mm) was purchased commercially from Sigma-Aldrich.

### Fabrication of graphene oxide (GO)/polycarbonate (PC) bilayer based infrared and sunlight-driven smart curtain

The schematic of fabrication process for GO/PC bilayer photoactuator is shown in Fig. 1. The home built setup of vacuum filtration was used to filter 1 ml of GO water dispersion through polycarbonate membrane followed by drying in ambient to form GO/PC bilayer structure. The extra part of PC membrane without GO coating was cut away to make curtain structure with diameter of about 28 mm.

### Fabrication of GO/PC bilayer based infrared actuated self-folding box

The GO/PC bilayer was fabricated using the above-mentioned procedure. This was followed by the fabrication of plastic sheet mask of plus sign shape with central portion dimensions of 1.0 cm × 1.0 cm while the dimension of each of the four wings was taken as 1.0 cm × 1.5 cm. The plastic sheet mask was then superimposed over fabricated bilayer structure such that extra portion was cut away resulting in the plus sign shaped GO/PC bilayer structure with the same dimensions as that of plastic sheet mask. The four wings were then folded once in upward direction to make their hinges around central portion, which is kept stationary under a small weight during the photoactuation measurements.

### Characterization and measurements

Raman spectrum (excited by 514 nm using Argon-ion laser) with 100X magnification at 5 mW power was obtained using Horiba Yuon Labram HR evolution Raman spectrometer. Thickness values of PC and GO film were determined through cross-sectional SEM using Zeiss Sipra 55 Field Emission Scanning Electron Microscope.IR absorption characteristics of GO films and PC membrane was determined by Vis-NIR spectrum which was obtained using UV-Vis-NIR spectrometer (Perkin Elmer Lambda 900).The variation of temperature on the GO face of actuator during actuation cycle was measured using IR thermometer (Fluke infrared thermometer). Photomechanical actuation measurements were done as per schematic shown in Fig. 3. The photoactuator structure comprised of GO/PC bilayer having circular dimensions with diameter of 28 mm and a thickness value of about 30 µm. This bilayer structure was illuminated with an IR light on GO face, keeping one of its diameter end fixed. The photoactuator deflects from its equilibrium position as a result of IR light illumination. The deflection from equilibrium position with time was recorded with a 12 mega pixel Nikon camera. The intensity of illumination was varied by varying the distance between actuator and the light source (780–1400 nm) with intensity values as 38, 59 and 106 mW/cm^2^ corresponding to the distance values between actuator and the light source as 25, 20 and 15 cm, respectively. The experiments were carried at RH of 48–51%.

## Electronic supplementary material

Supplementary Information

Supplementary Movie S1

Supplementary Movie S2

Supplementary Movie S3

Supplementary Movie S4
