# Supplementary material for: Sunlight-driven eco-friendly smart curtain based on infrared responsive graphene oxide-polymer photoactuators
Source: Sci Rep. 2018 Feb 27;8:3687. doi: 10.1038/s41598-018-21871-3 (PMC5829250; doi:10.1038/s41598-018-21871-3)
Supplement: Supplementary file 1 — Supplementary Information [file 41598_2018_21871_MOESM1_ESM.pdf]

Supplementary Information

Sunlight-driven eco-friendly smart curtain based on infrared responsive graphene oxide-polymer photoactuators

Leeladhar[1,2], Parul Raturi[1] and J.P. Singh*[1]

[1]*Department of Physics, Indian Institute of Technology Delhi, Hauz Khas, New Delhi 110016, India.*

[2]*Solid State Physics Laboratory, Lucknow Road, Timarpur, Delhi 110054, India.*

**Address correspondence to* jpsingh@physics.iitd.ac.in

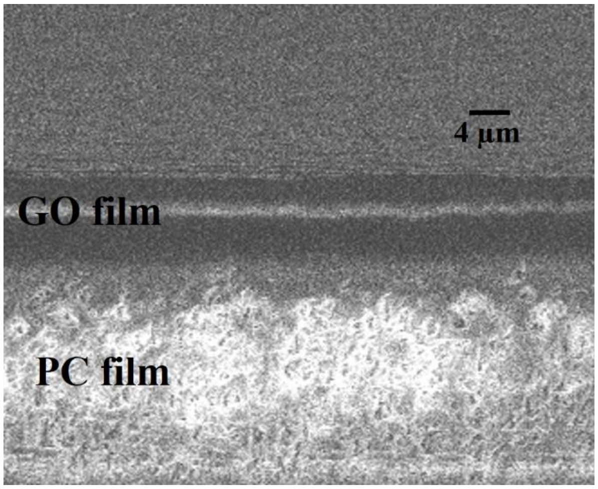

Supplementary Figure S1. Cross-sectional SEM image of GO/PC bilayer structure (GO film thickness~ 8µm and PC film thickness~22µm)

Supplementary Note S1

Calculation of bending curvature from bending deflection

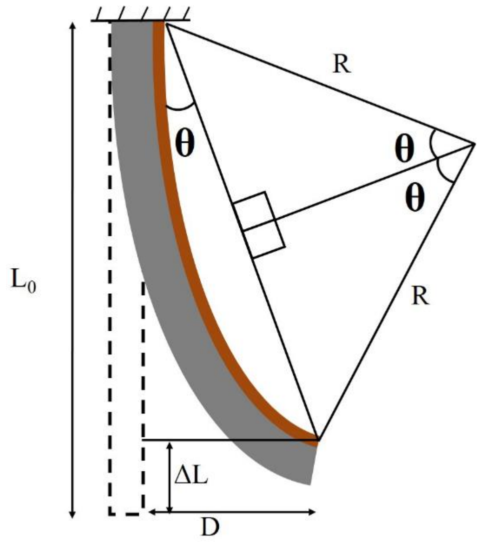

Figure S2. Geometry of GO/PC bilayer photoactuator in bending state.

Bending curvature is inverse of redius of curvature and using Figure S2, it is given by following expression.

$$\rho = \frac{2\sin\theta}{\sqrt{(L^2 + D^2)}}$$

$$\rho = \frac{2\sin\left(\tan^{-1}\frac{D}{L}\right)}{\sqrt{(L^2 + D^2)}} \qquad ------(1)$$

Where

D = bending deflection.

L_0 = original length of photoactuator structure when it is in normal state.

ΔL = change in length of photoactuator structure when it is in actuated state.

L = ($L_0 - \Delta L$) = effective length of photoactuator structure when it is in actuated state.

So, for illumination intensity of 106 mW/cm^2 , by making use of equation (1) and values of all parametrs as D = 12 mm, L_0 = 28mm, ΔL~ 4mm, bending curvature is found to be 0.33 cm^{-1}.

Supplementary Movies

Supplementary Movie S1. Photomechanical actuation response of GO/PC bilayer photoactuator under infrared light with illumination intensity of $106mW/cm^2$.

Supplementary Movie S2. Infrared light ($106mW/cm^2$) actuated curtain.

Supplementary Movie S3. Sunlight-driven smart curtain with illumination intensity of $100mW/cm^2$.

Supplementary Movie S4. Infrared light ($106mW/cm^2$) actuated self-folding box.
